# The PYK2 inhibitor PF-562271 enhances the effect of temozolomide on tumor growth in a C57Bl/6-Gl261 mouse glioma model

**DOI:** 10.1007/s11060-023-04260-3

**Published:** 2023-02-15

**Authors:** Jescelica Ortiz-Rivera, Rebeca Nuñez, Yuriy Kucheryavykh, Lilia Kucheryavykh

**Affiliations:** Department of Biochemistry, School of Medicine, Universidad Central de Caribe, Bayamon, PR 00956 USA

**Keywords:** GBM, Temozolomide, PF-562271, FAK, Pyk2

## Abstract

**Background:**

The development of resistance to temozolomide (TMZ), a standard chemotherapeutic, limits the effective treatment of glioblastoma (GBM). Focal adhesion kinase (FAK) and proline rich tyrosine kinase 2 (Pyk2) regulate proliferation and invasion of GBM cells. We found that TMZ activates FAK and Pyk2 signaling in GBM. We hypothesized that pharmacological inhibitors of Pyk2/FAK together with TMZ can enhance the inhibitory effect of TMZ on tumor growth and dispersal and improve the treatment outcome.

**Methods:**

Primary human GBM cell cultures and a C57Bl/6-GL261 mouse glioma implantation model were used. Pyk2 (Tyr579/580) and FAK (Tyr925) phosphorylation was analyzed by western blotting. Viability, cell cycle, migration, invasion and invadopodia formation were investigated in vitro. Animal survival, tumor size and invasion, TUNEL apoptotic cell death and the Ki67 proliferation index were evaluated in vivo upon treatment with TMZ (50 mg/kg, once/day, orally) and the Pyk2/FAK inhibitor PF-562271 (once/daily, 50 mg/kg, orally) vs. TMZ monotherapy.

**Results:**

In vitro studies revealed significantly reduced viability, cell cycle progression, invasion and invadopodia with TMZ (100 µM) + PF-562271 (16 nM) compared with TMZ alone. In vivo studies demonstrated that combinatorial treatment led to prominent reductions in tumor size and invasive margins, extensive signs of apoptosis and a reduced proliferation index, together with a 15% increase in the survival rate in animals, compared with TMZ monotherapy.

**Conclusion:**

TMZ + PF-562271 eliminates TMZ-related Pyk2/FAK activation in GBM and improves the treatment efficacy.

**Supplementary Information:**

The online version contains supplementary material available at 10.1007/s11060-023-04260-3.

## Introduction

Glioblastoma (GBM) is one of the most aggressive brain tumors and has a poor prognosis. The overall survival in most patients does not exceed 2 years [1]. The development of resistance to temozolomide (TMZ), a standard-of-care chemotherapeutic for newly diagnosed GBM [2], limits effective treatment. GBM tumors may acquire TMZ resistance through alterations in their expression of DNA repair enzymes [3] but also through modulations in cell signaling pathways involved in the regulation of the cell cycle, metabolism and DNA repair machinery [4–7]. Therefore, targeting alternative pathways that are affected or altered by TMZ treatment can improve treatment outcomes.

Focal adhesion kinase (FAK) and proline-rich tyrosine kinase 2 (Pyk2) are nonreceptor tyrosine kinases that link extracellular signaling to the regulation of cell proliferation, migration, and survival [8]. Increased FAK activity has been correlated with increased cell proliferation and motility, playing a key role in tumor progression [8–11]. Our previous studies demonstrated that FAK and Pyk2 are activated in GBMs, and the phosphorylation of FAK and Pyk2 correlated with GBM cell proliferation and invasion [12, 13]. Additionally, we demonstrated that cytokines and chemokines released by tumor infiltrating myeloid cells (TIMs) activate Pyk2 and FAK in GBM cells to promote proliferation and dispersal [13].

Here, we showed that TMZ treatment activated FAK and Pyk2 signaling in GBM cells. We hypothesized that pharmacological inhibitors of Pyk2/FAK together with TMZ can enhance the inhibitory effect of TMZ on GBM tumor progression and improve the treatment outcomes. In vitro approaches using primary human GBM cell cultures with different levels of FAK and Pyk2 expression combined with a mouse C56Bl/6-GL261 glioma implantation model were used in the study. We demonstrated that combined treatment significantly reduced the viability, proliferation and invasion of GBM cells compared with TMZ monotherapy and decreased tumor growth and animal mortality. Since TIMs indicate a significant impact on Pyk2/FAK signaling activation in GBM, all in vitro studies were performed using microglia-conditioned medium (MCM) to mimic the tumor microenvironment.

## Methodology

### Cell culture

Primary human GBM CL-2 and CL-3 cells, developed from GBM specimens, were previously characterized [13]. The mouse GL261 glioma cell line was obtained from the National Cancer Institute (Frederick, MD, USA). Human HMC3 and mouse SIM-A9 microglia was obtained from ATCC (#CRL-3304, #CRL-3265, ATCC, Rockefeller, VA, USA). Cells were cultured in Dulbecco’s modified Eagle’s medium (DMEM, #D7777, Sigma-Aldrich, Saint Louis, MO, USA) with 10% fetal bovine serum (#35-010-CV, Corning, Inc., AZ, USA), 50 U/mL penicillin, and 50 µG/mL streptomycin. Cells were cultivated for no more than 16 passages.

### Western blot analysis

Cell lysates (20 µg), separated by 10% SDS‒PAGE, were transferred to PVDF membranes and probed with anti-phospho-Pyk2 (Tyr 579/580) (#44636G, Thermo Fisher Scientific, Invitrogen, MA, USA), anti-Pyk2, anti-phospho-FAK (Tyr 925), anti-FAK, anti-BCL2 and anti-Cyclin D1 (#3480, #3284, #3285, #3498, #55506 Cell Signaling, Danvers, MA, USA) antibodies.

Detection was performed with enhanced chemiluminescence (#34075, SuperSignal West Dura Extended Duration Substrate; Pierce, Rockford, IL, USA). The signal intensity was measured using a gel documentation system (Versa Doc Model 1000, Bio-Rad, Hercules, CA, USA). Research Resource Identifiers for cells and antibodies are presented in Online Recourse 1.

### Microglia-conditioned medium

HMC3 microglia and GBM cells were cocultured at a 1:1 ratio for 24 h. The obtained MCM was centrifuged and used for experiments.

### Cell viability assays

Cells were incubated with MCM with 100 µM TMZ (#T2577, Sigma-Aldrich, Saint Louis, MO, USA), 16 nM of the Pyk2/FAK inhibitor PF-562271 (#202228, MedKoo Bioscience, Morrisville, NC, USA) or TMZ + PF-562271 at concentrations given alone for 72 h. The number of live and dead cells was determined by the Live/Dead Viability/Cytotoxisity Kit (#L3224, Invitrogen, MA, USA), based on calcein (green) and ethidium homodimer-1 (red) staining of live and dead cells, respectively.

### Intracranial glioma implantation

C57Bl/6 mice were obtained from the Jackson Laboratory. Under isoflurane anesthesia, GL261 cells were implanted into the cerebral hemisphere of 12–20 week-old mail and female mice at stereotaxic coordinates of 2 mm lateral and 1 mm caudal to bregma. One microliter of cell suspension (2 × 10^4^ cells/μL in PBS) was delivered at a depth of 3 mm.

### Oral gavage

Beginning on the 5th day after tumor implantation, animals received 2 weeks of treatment through oral gavage without interruptions: vehicle (100 µL of 5% DMSO + 10% hydroxypropyl-β-cyclodextrin), TMZ (50 mg/kg), PF-562271 (50 mg/kg), as described previously [14, 15], or TMZ with PF-562271 simultaneously at concentrations given alone once/day.

### Purification of glioma cells from tumors

Tumors were homogenized using nonenzymatic cell dissociation (#130-096-730, Sigma-Aldrich, St. Louis, MO, USA) and glioma cells were purified using Percoll (#E-0414, Sigma-Aldrich) gradients.

### Tumor size evaluation

Frozen 15 µm brain sections encompassing tumors were stained with hematoxylin & eosin (H&E). Tumor size was calculated as the sum of tumor area x section thickness in all sections encompassing tumor. Invasion margin is measured as a deepest point of invasion from the tumor invasion front.

### Immunofluorescence imaging

Twenty-five micrometer frozen sections of tumors were processed with anti-Ki67 antibody (#12075, Cell Signaling, Danvers, MA, USA), followed by Taxes Red-conjugated IgG (#TI-1000, Vector Laboratories, Burlingame, CA, USA). A TUNEL assay kit (#ab206386, Abcam, Boston, MA, USA) was used according to the manufacturer’s protocol. Sections were visualized using an Olympus Fluoview FV1000 confocal microscope (Olympus, Japan) and processed using ImageJ software (http://imagej.nih.gov/ij, version 1.54b, assessed on 08 January 2023).

### Cell cycle analysis

Cells were fixed with 70% ethanol, resuspended in the Guava® Cell Cycle Reagent Kit (#4500-0220, Luminex Corporation, Hayward, CA, USA) and analyzed with the Guava easyCyte flow cytometer (Luminex Corporation) and the InCyte software module (Luminex Corporation).

### Invasion and migration assays

Invasion assays were performed using Fluoroblok inserts (8 μM pore size, VWR Scientific, Batavia, IL, USA) coated with 30 μL of Matrigel Matrix (#354263, BD Biosciences, Bedford, MA). Serum-starved glioma cells (30,000) were placed on top of the membrane, and microglia (30,000 cells) were placed in the lower compartment. After 16 h, the cells were fixed and stained with propidium iodide. Cells that invaded to the lower compartment were counted. For migration assays, Matrigel was not used.

### Invadopodia assay

Cells were plated on fluorescein-conjugated gelatin-coated coverslips (#G13187, Invitrogen) for 16 h, fixed with 4% PFA, stained with phalloidin–tetramethyl–rhodamine (#P1951, Sigma-Aldrich) and DAPI (#D9542, Sigma-Aldrich), visualized using an Olympus Fluoview FV1000 confocal microscope (Olympus) and analyzed using ImageJ software. For the quantification of invadopodia formation (IF), cells forming invadopodia were counted and normalized to the number of nuclei in each image. For quantification of invadopodia activity (IA), the gelatin fraction was normalized to the number of nuclei.

### Survival analysis

Upon glioma implantation, the mice were inspected daily, and those with body weight loss of 15%, decreased activity/responsiveness, and signs of neurological disorders were euthanized. The time between tumor development and animal death was recorded. Comparison of survival curves was performed using the log-rank (Mantel‒Cox) test.

### Statistical analysis

Statistical probability was calculated using GraphPad Prism 1.0 software. One-way ANOVA was used for comparisons between groups, and two-way ANOVA was used for comparisons among groups under different conditions. Statistical significance (p ≤ 0.05) was determined by Dunnett’s multiple comparisons test and unpaired t tests with Welch’s correction.

## Results

### TMZ increases Pyk2 and FAK phosphorylation in glioma cells

**Fig. 1:**
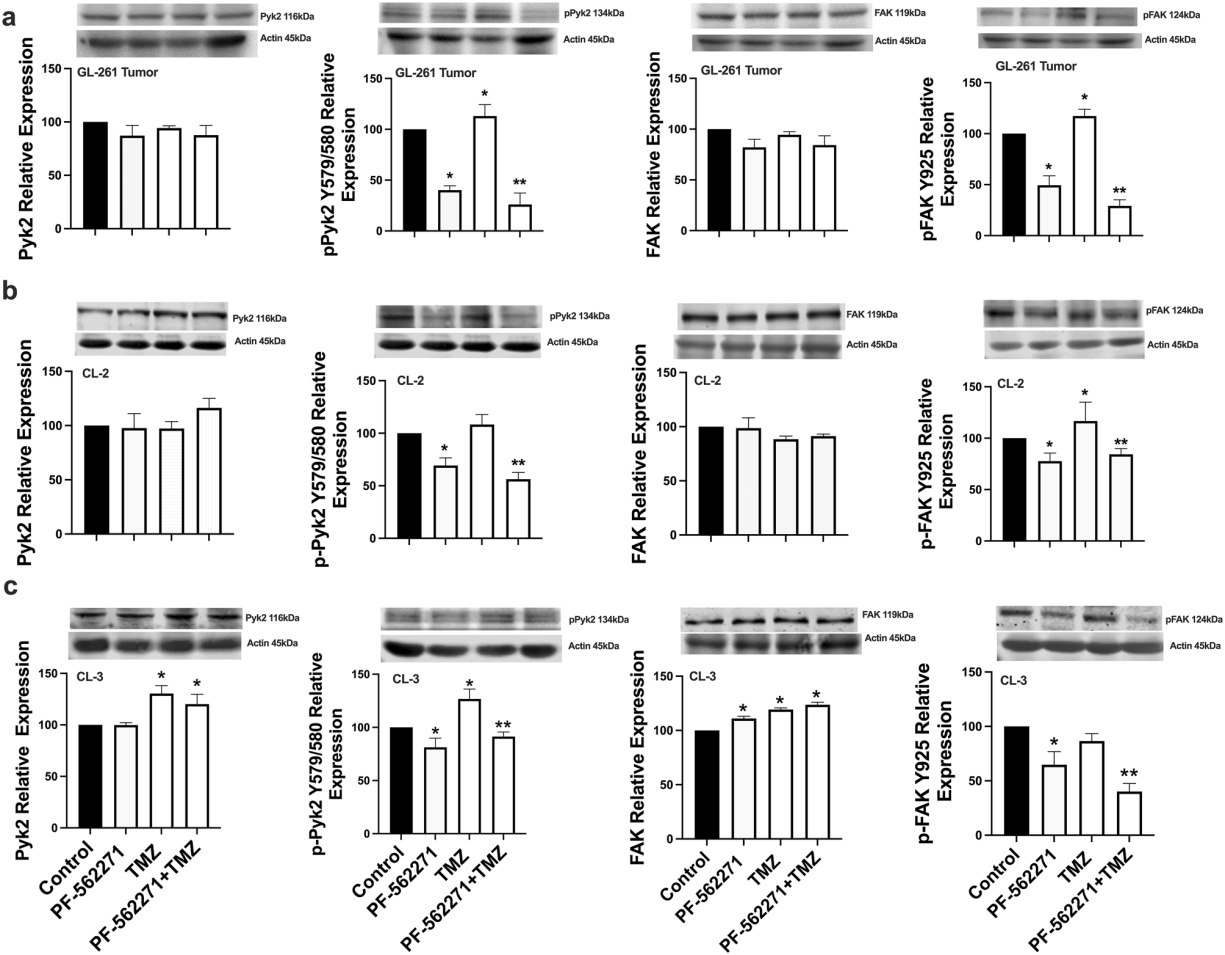
PF-562271 reverses the stimulatory effect of TMZ on Pyk2 and FAK phosphorylation in glioma cells. Representative western blots and quantifications for average levels of total and phosphorylated Pyk2 and FAK protein expression for each treatment group relative to control are presented for glioma cells purified from tumors generated in the C57BL/6-GL261 glioma mouse model (**a**) and for primary human GBM cultured CL-2 (**b**) and CL-3 cells (**c**). Images are presented for the control, PF-562271, TMZ, and PF-562271 combined with TMZ groups. Tumors were analyzed after 14 days of treatment, given beginning the 5th day after tumor implantation. Six animals per experimental group was used. In vitro data are shown for 24 h of treatment in each experimental group. Actin was used as a loading control. Mean ± S.E. and significant differences from the control (*) or TMZ groups (**) are shown (p < 0.005). N = 6

Western blot analysis was performed to evaluate Pyk2 and FAK signaling in implantation-generated tumors. As Pyk2 and FAK are also expressed in TIMs [16, 17], glioma cells were purified from total tumors. 20% upregulation of pPyk2 (579/580) and pFAK (925) in glioma cells upon TMZ treatment, without significant changes in total Pyk2 and FAK protein expression, was identified (Fig. 1a). Phosphorylated Pyk2 and FAK were reduced by 50% in animals that received PF-562271 and by 70% with TMZ + PF-562271 treatment compared with the control.

Primary human GBM CL-2 and CL-3 cells, characterized in a previous publication [13] and in Online Resource 2, were used for in vitro analysis. CL-2 is characterized with positive expression of neurofibromatosis 1 (NF1) and O[6]-methylguanine-DNA methyltransferase (MGMT) and with high expression of FAK with low FAK phosphorylation, while CL-3 has NF1-negative, MGMT positive phenotype and has moderate basal expression of FAK and Pyk2. As cytokines released from TIMs affect Pyk2 and FAK activation in glioma cells [12, 13] and in an attempt to mimic the tumor microenvironment, studies were performed in MCM. CL-2 demonstrated 30% upregulation of pFAK(925), while CL-3 upregulated pPyk2(Tyr579/580) by 30% in response to TMZ treatment (Fig. 1b, c). No significant differences in total Pyk2 and FAK protein expression between treatments were detected in CL-2; however, 30% upregulation of total Pyk2 and 20% upregulation of total FAK expression were detected in CL-3 in response to TMZ treatment. Variability in treatment responses between cell lines is likely related to the loss of NF1 and high basal FAK expression in CL-3, contrary to CL-2 and the mouse GBM model. PF-562271 inhibited the basal phosphorylation of Pyk2 and FAK by 38% and 35%, respectively, in CL-2 cells and by 25% and 35%, respectively, in CL-3 cells and reversed the stimulatory effect of TMZ in both cell lines. Overall, TMZ increased Pyk2 and FAK signaling in all investigated models, while PF-562271 reversed this effect.

### Combined TMZ and PF-562271 treatment reduces cell viability and proliferation in primary GBM human cell lines compared to TMZ monotherapy

**Fig. 2:**
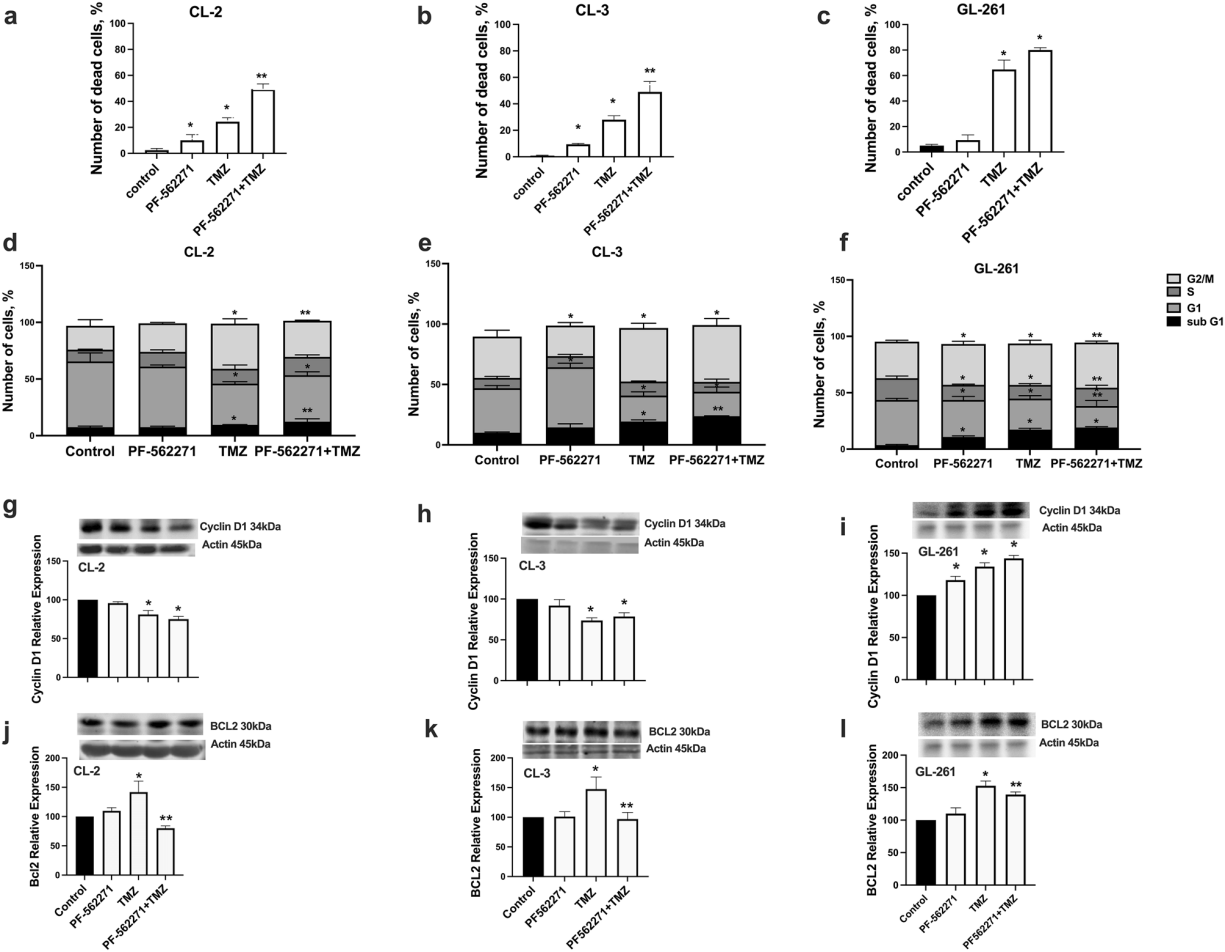
TMZ and PF-562271 combinatorial treatment reduced cell viability in primary human glioma cell lines. **a**–**c** The number of dead cells was calculated as a percent of dead cell relative to total number of cells using the viability assays after 72 h of treatment with vehicle (control), PF-562271, TMZ, and PF-562271 + TMZ in CL-2, CL-3 and GL261 cell lines. Live and dead cells were quantified based on calcein and ethidium homodimer-1 staining for live and dead cells. **d**–**f** Cell cycle analysis was performed for CL-2, CL-3 and GL261 cells using flow cytometric evaluation of propidium iodide as a nuclear marker. The percentage of cells in the G0/G1, S and G2/M phases was determined based on DNA content. Bar graphs represent the total distribution of cells at different phases of the cell cycle. **g**–**l** Relative expression of Bcl-2 and cyclin D1 in CL-2 (**g**, **j**), CL-3 (**h**, **k**) and GL261 (**i**, **l**) evaluated by western blots and analyzed as fold change relative to control are presented. Actin was used as a loading control. N = 4. Mean ± S.E. and significant differences from the control (*) and (**) from TMZ are shown (p < 0.05)

Viability assays detected 11% and 10% of dead cells upon 72 h of PF-562271 treatment, 34% and 29% with TMZ treatment and 48% and 55% with PF-562271 + TMZ treatment (Fig. 2a, b, Online Recourse 3) in CL-2 and CL-3 respectively. GL261 cells exhibited 10% dead cells in PF-562271, 60% in TMZ and 80% in combinatorial treatments (Fig. 2c). These results indicate that the cytotoxic effect of combinatorial treatment was 30% greater in CL-2 and GL261, and 80% greater in CL-3, compared with that of TMZ monotherapy, suggesting the involvement of Pyk2 and FAK in survival mechanisms, limiting TMZ cytotoxicity.

Cell cycle analysis demonstrated different basal cell cycle distribution in investigated cell lines with up to 50% of cell in G2/M in CL-2 vs. 21% and 32% in CL-2 and GL261 correspondingly (Fig. 2d–f, Online Recourse 4). No significant modifications in the cell cycle in CL-2 and CL-3 cells upon 72 h of treatment with PF-562271 compared with the control was detected, while reduction of G1 population and increase in G2/M and sub-G1, indicative for cell cycle arrest, was detected in GL261. In all cell lines accumulation of cells in G2/M and sub-G1 phases was detected after TMZ treatment. However, combinatorial treatment resulted in a further 20% increase in the sub-G1 population in CL-2 and CL-3, compared with TMZ monotherapy, indicating direction of cells to apoptosis upon cell cycle arrest in both cell lines. This effect was combined with restriction of the S to G2/M transition in CL-2, but not in CL-3. Increased accumulation of cells in G2/M and sub-G1 and reduction in G1 was detected GL261 cells in combinatorial treatment compared with TMZ. The results indicate that combinatorial treatment increases cytotoxic effects compared with TMZ monotherapy in all investigated cell lines and restricts the S to G2/M transition in CL-2 and GL261.

**Fig. 3:**
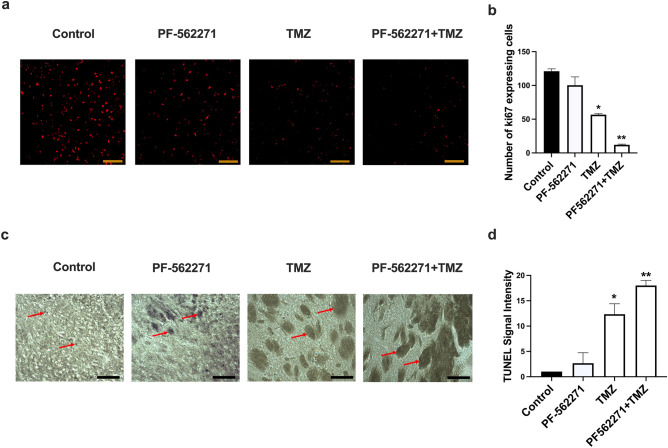
TMZ and PF-562271 combinatorial treatment reduced tumor cell proliferation and increased apoptosis in the C57BL/6-GL261 mouse glioma implantation model compared with TMZ monotherapy. Immunofluorescence confocal images of tumors (**a**) and quantification (**b**) of cells expressing the Ki67 marker are presented for animals that received vehicle, TMZ, PF-562271, and combined TMZ and PF562271 treatment for 7 days, beginning the 10th day after tumor implantation. Number of Ki67-positive cells in a field of view is presented. **c**, **d** Immunohistochemical images of tumors and quantification of the induction of apoptosis using TUNEL assays for animals that received vehicle, TMZ, PF-562271, and combined treatment with TMZ and PF562271. Arrows indicate the TUNEL stained individual cells or cells aggregations. TUNEL signal intensity was quantified as average gray measurements, normalized to the background, with use of ImageJ program. Data are presented as relative to control. Scale bar is 100 µm. Mean ± S.E. and significant differences from the control (*) or TMZ group (**) are shown (p < 0.005). N = 6

PF-562271 treatment did not significantly affect Bcl-2 and Cyclin D1 expression compared with the control in all cell lines (Fig. 2j–l). However, 50% upregulation of Bcl-2 was observed in response to TMZ, which was reversed with combinatorial TMZ + PF-562271 treatment in all cell lines. Together with data obtained from viability and cell cycle analysis, these results indicate that TMZ exacerbates antiapoptotic signaling in GBM cells, which is reversed by Pyk2/FAK inhibition. The 25% decrease in Cyclin D1 expression upon TMZ treatment reflects the reduction in the number of cells entering mitosis in CL-2 and CL-3 (Fig. 2g, h). The deeper decrease in Cyclin D1 up to 30% in CL-2 in combinatorial treatment, but not in CL-3, correlated with the cell cycle analysis data and indicated the involvement of the Pyk2/FAK signaling component in the TMZ cytostatic mechanism. Contrary, up-regulation of Cyclin D1 in all treatment groups was detected in GL261. Taking in account high susceptibility of GL261 cells to TMZ and PF-562271, this might indicate different signaling mechanisms of TMZ and PF-562271-driven cell death in this cell line.A C57BL/6-GL261 glioma mouse model was used to assess the cytotoxic effect of PF-562271 + TMZ combinatorial treatment in vivo. Immunocytochemical detection of Ki67 identified no significant decrease in the number of cells expressing Ki-67 in animals that received PF-562271 beginning 10^th^ day after implantation, for 7 days, compared with the control. However, 52% and 97% reductions were detected in tumors from TMZ and combinatorial treatment, respectively (Fig. 3a, b). TUNEL assays revealed that in animals that received PF-562271, 7% of tumor tissue showed signs of apoptosis, 12% in the TMZ group and 17% in the PF-562271 + TMZ group (Fig. 3c, d). Overall, although TMZ induces cell death and reduces proliferation in GBM cells, this effect is significantly exacerbated when TMZ treatment is combined with Pyk2/FAK inhibition.

### TMZ and PF-562271 combined treatment reduces invasiveness in GBM cells

**Fig. 4:**
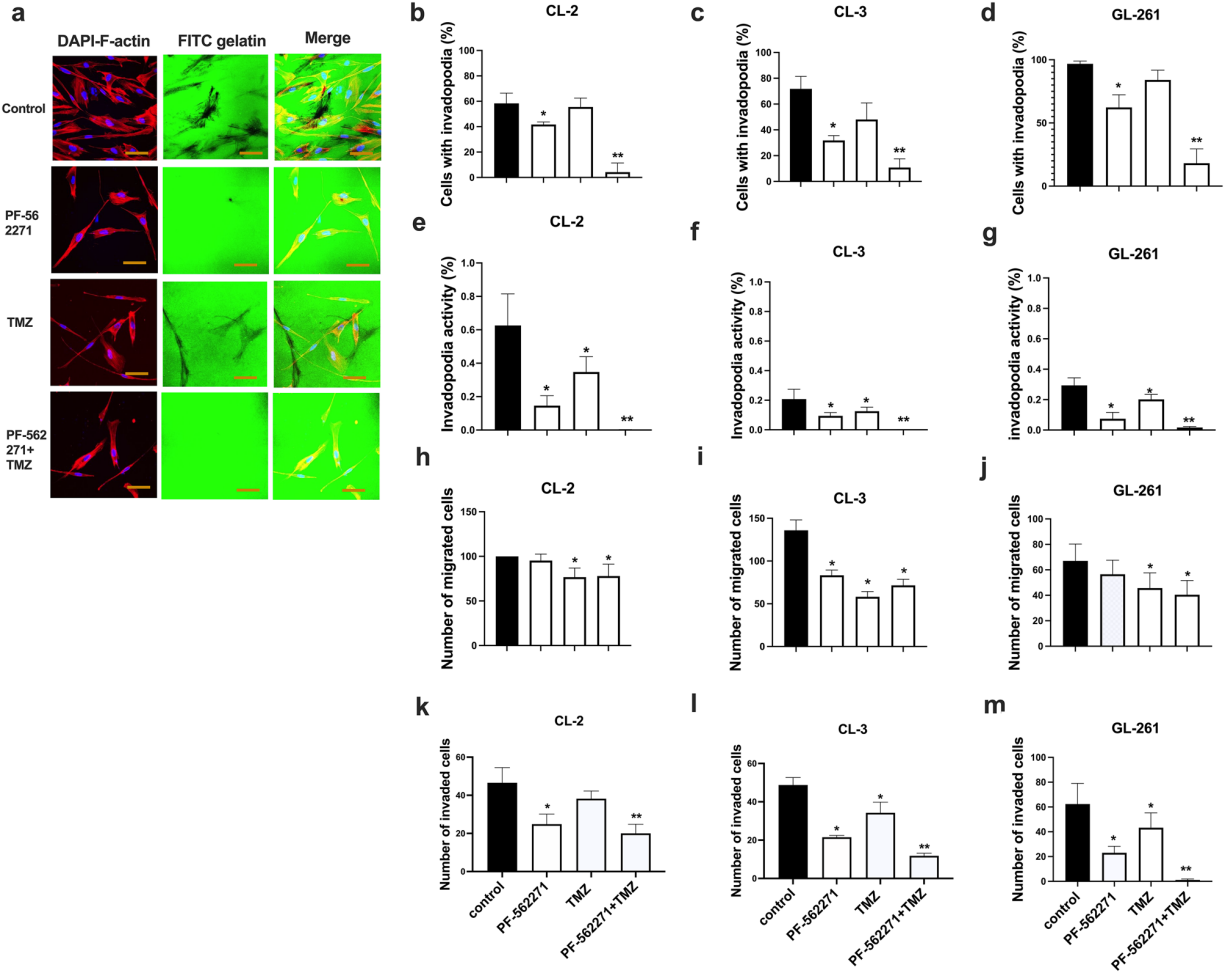
TMZ and PF-562271 combinatorial treatment decreases functional invadopodia formation, migration and invasion in primary human GBM cells. Invadopodia formation assays, performed for CL-2, CL-3 and GL261 cells, are presented as confocal images (confocal images for CL-2 are presented in (**a**), images for CL-3 and GL261 are presented in Online Recourse 5) and calculations of the number of cells forming invadopodia (**b**–**d**), together with calculations of area fraction gelatin matrix (invadopodia activity, **e**–**g**) for cells treated with vehicle, TMZ, PF-562271, and TMZ and PF-562271 in combination. Study duration was 16 h. Cells with invadopodia are presented as a ratio of cells forming invadopodia to the total number of nucleuses in each image. Invadopodia activity is presented as a ratio of area fraction to the total area and normalized to the total number of cells in each image. F-actin, stained with rhodamine–phalloidin (red), FITC-conjugated gelatin (green), and DAPI, used for nuclei staining (blue), are shown. Degraded areas of FITC-labeled gelatin are shown as black patches. Transwell migration (**h**–**j**) and invasion (**k**–**m**) assays were performed for CL-2, CL-3 and GL261 cells. The relative number of migrating and invading glioma cells, compared to control, is presented. Scale bar: 60 µm. Mean ± S.D. with significant differences from controls (*) and TMZ (**) are shown (p < 0.05). N = 5

Invadopodia assays were performed to evaluate the extracellular matrix degradation capacity in investigated cell lines. The number of cells that formed invadopodia (IF) and gelatin matrix degradation (IA) were evaluated (Fig. 4a–g, Online Recourse 5). TMZ monotherapy did not significantly affect IF but resulted in a 40% reduction in IA in all cell lines. PF-562271 reduced the IF by 18%, 50% and 34% and reduced the IA by 73%, 67% and 76% compared with the control in CL-2, CL-3 and GL261, respectively. However, combinatorial treatment decreased the IF by 85%, 81% and 82% compared with the control and by 82%, 71% and 79% compared with TMZ monotherapy in CL-2, CL-3 and GL261, respectively, while it decreased the IA by 98%, 95% and 94% compared with the control and by 90%, 92% and 91% compared with TMZ monotherapy in CL-2, CL-3 and GL261.

Migration assays identified no effect on cell migration in CL-2 and GL261 upon PF-562271 treatment, while TMZ monotherapy decreased cell migration by 20% and 33% respectively with no additional effect with PF-562271 + TMZ combinatorial treatment (Fig. 4h, j). In contrast, in CL-3, significant 30% and 45% reductions in cell migration were observed with the PF-562271 and TMZ treatments (Fig. 4i), with no additive effect with the PF-562271 + TMZ combinatorial treatment.

Invasion assays demonstrated (Fig. 4k–m) a 50% reduction in invasion after PF-562271 treatment in CL-2 and CL-3 cells and 63% reduction in GL261. TMZ inhibited cell invasion in CL-3 and GL261 but not in CL-2; however, combinatorial treatment resulted in 48%, 66% and 97% reductions in invasion compared with the TMZ in CL-2, CL-3 and GL261, respectively. Thus, TMZ alone inhibits GBM cell migration and invasion, but combinatorial inhibition of Pyk2 and FAK signaling dramatically reduced the extracellular matrix degradation of GBM cells and, consequently, their invasion.

### Combined TMZ and PF-562271 treatment reduces tumor growth and animal survival in a C57BL/6-GL261 mouse model

**Fig. 5:**
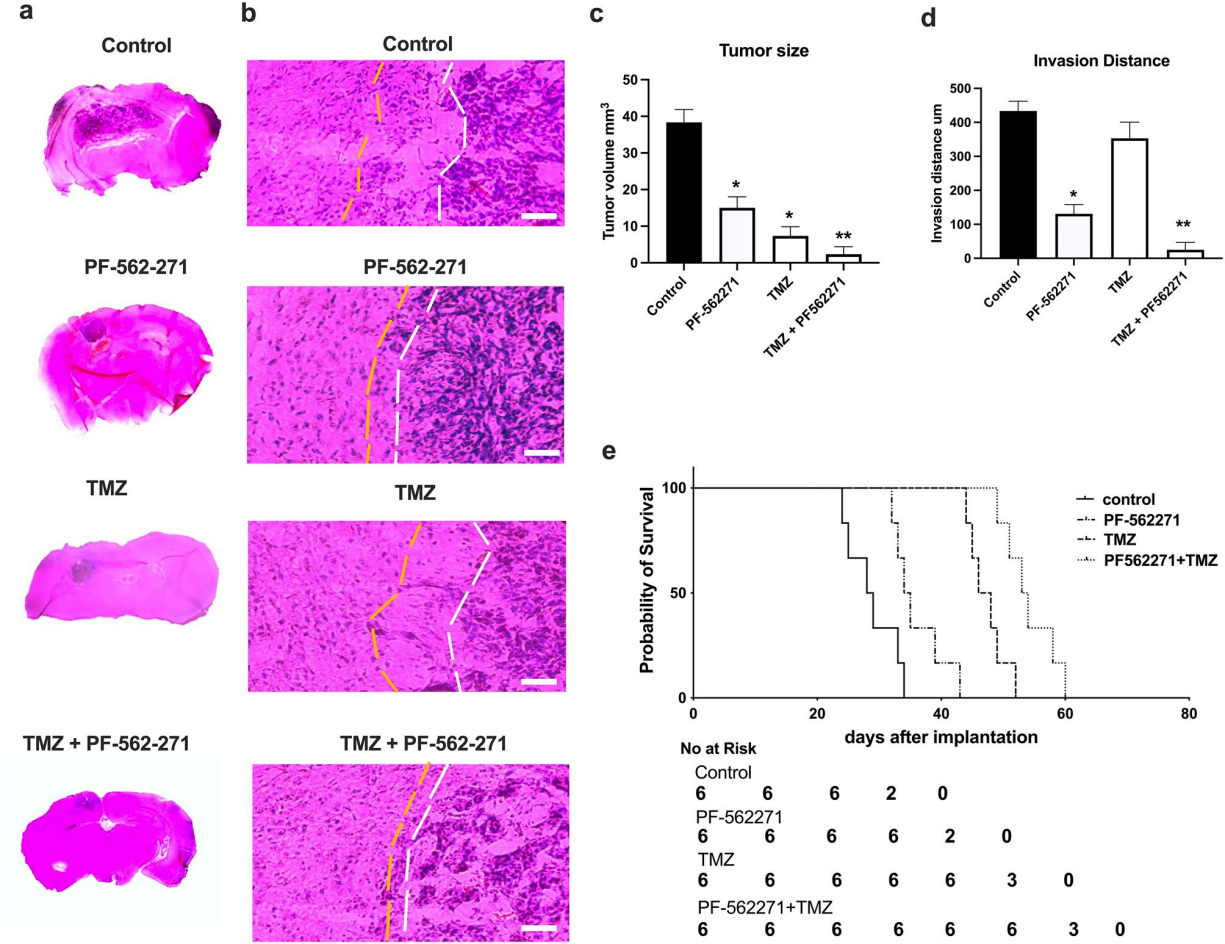
TMZ combined with PF-562271 reduces tumor growth and invasion margins and increases animal survival rates in a C57BL/6-GL261 mouse glioma implantation model compared with TMZ monotherapy. Hematoxylin and eosin staining of mouse brain slices encompassing implanted tumors (**a**, **c**) and quantification of tumor size (**b**) and invasion distance (**d**) in animals that received vehicle, PF-562271, TMZ, and PF-562271 + TMZ combinatorial treatments for 14 days after tumor implantation. Tumor size evaluation was performed immediately after treatment course termination. **e** Kaplan‒Meier estimates of overall survival probability for animals that received vehicle, PF-562271, TMZ and PF-562271 + TMZ combinatorial treatments. Six animals per group were used in all presented studies. Curve comparison was performed using the log-rank (Mantel‒Cox) test (p < 0.05). The results are presented as the mean ± S.D. with significant differences from control (*) and TMZ (**) (p < 0.05). Scale bar: 100 µm

Immunohistochemical evaluation of tumor size was performed to evaluate effect of PF-562271 on tumor growth. As shown in Fig. 5a, b, a 55% reduction in tumor volume was detected in PF-562271-treated animals, and an 80% reduction was detected with TMZ treatment compared with the control, as detected upon 14 days of treatment. A 77% reduction in the tumor invasion margin was found upon PF-562271 treatment, while in TMZ monotherapy, the invasion distance did not differ from the control (Fig. 5c, d). Animals that received combinatorial treatment developed significantly smaller tumors compared with those of PF-562271 or TMZ monotherapy, with a 95% reduction in invasion margins compared to that of TMZ. The results indicate that PF-562271, used in combination with TMZ, enhances the effect of TMZ on tumor growth inhibition and invasiveness. Survival analysis further demonstrated that despite no significant difference in median survival between the control and PF-562271 treatment groups (28.5 vs. 34.5 days), a 15% increase in median survival in PF-562271 + TMZ treatment (53 days) vs. TMZ monotherapy (46 days) was detected.

## Discussion

The mechanism of resistance to TMZ varies among tumors and differs in terms of innate or acquired resistance, including alterations in DNA repair genes [18–20]. Here, we identified enhanced Pyk2 and FAK signaling in response to TMZ treatment, which contributes to TMZ resistance. The use of Pyk2/FAK inhibition upon TMZ treatment may improve current treatment protocols.

We demonstrated that PF-562271 enhanced TMZ cytotoxicity in GBM cells (Fig. 2). Cell cycle analysis revealed that TMZ treatment increased G2/M and sub-G1 populations together with up-regulation of Bcl2 expression in all cell lines, indicative for cell cycle arrest (Fig. 2). GBM cell resistance to therapy has been attributed to the overactivation of G2 checkpoint kinases [21], which allows cells to repair their DNA and prevent mitotic catastrophe [22, 23]. Combinatorial treatment further increased sub-G1 population in GBM cells with increase of G2/M population in CL-2 and GL261, without significant effect on the G2/M cell population in CL-3, and with substantial decrease of Bcl2 expression, compared with TMZ in all cell lines. Prominent accumulation of cells in G2/M in GL261 in combinatorial treatment indicate higher sensitivity of these cells to TMZ and PF-562271, which is also confirmed with viability assays. Up-regulation of Bcl2 in response to treatment with TMZ was recently demonstrated [24]. Bcl-2 has been linked to resistance against adjuvant treatment [25], and our data suggest that PF-562271 counteracts antiapoptotic mechanisms activated by TMZ in GBM cells. Additionally, accumulation of cells in G1in CL-2, accompanied with increase of sub-G1 population in combinatorial treatment, compared with TMZ, indicates that Pyk2 and FAK signaling are involved in the S/G2 transition, as previously shown [13]. As GBMs are intrinsically resistant to apoptosis [3], impairment of the S/G2 transition may increase the cytostatic and cytotoxic effects of TMZ. Impairment of the S/G2 transition was not observed in CL-3, which is NF1 negative. Loss of NF1 leads to sustained activation of RAS-GTP, activation of the RAS/RAF/MAPK, Akt and FAK signaling pathways, together with up-regulation of heat shock factor 1(HSF1), and contributes to increased cell proliferation, survival and therapy resistance [26–30]. Treatment with FAK and MAP-ERK inhibitors was shown to sensitize NF1^−^ cancers to chemo- and targeted therapy [26, 31, 32]. Despite signaling mechanisms of sensitizing to TMZ with use of PF-562271 may be different in NF1^+^ and NF1^−^ GBMs, our study demonstrated the efficacy of combinatorial treatment in cell lines of both phenotypes. Although the number of apoptotic GL261 cells in combinatorial treatment did not significantly differ from TMZ treatment, a significant reduction of total cell count, as defined by viability and cell cycle analysis, indicates involvement of another then apoptosis cell death mechanisms. The role of autophagy instead of apoptosis in GBM in response to TMZ-induced cell death was previously described [33, 34]. Additionally, up-regulation of CyclinD1, that was detected in GL261 cells upon treatment with TMZ and PF-562271, correlated with up-regulation of cell death. Some reports demonstrated that cyclin D1 overexpression may enhance the caspase-dependent and induced endoplasmic reticulum stress-mediated apoptosis in cancers [35, 36]. However, more detailed studies of the effect of Cyclin D1 overexpression on treatment susceptibility in specific GBM phenotypes are needed.

Invadopodia are implicated in ECM degradation and cancer cell invasion [37]. Although combinatorial treatment did not affect migration, compared with TMZ monotherapy, a drastic reduction in IA and invasion with combinatorial treatment, compared with TMZ monotherapy, was detected in all cell lines (Fig. 4). TMZ did not affect the formation and activity of invadopodia, which is consistent with literature reports, demonstrating that GBM cells increase IF upon TMZ treatment, and cells that survive TMZ treatment acquire a more pro-invasive phenotype [38, 39]. Pyk2 and FAK were previously shown to regulate invasion [8, 12], and Pyk2/FAK inhibitors could prevent tumor cell dispersal from the tumor core upon TMZ treatment. Additionally, PF-562271 promoted strong down-regulation of IA, migration and invasion capabilities in CL-3, compared to CL-2 and GL261, that might be related to loss of NF1 and susceptibility to FAK activation [29].

In vitro studies’ conclusions are further supported by in vivo studies, which demonstrated that despite TMZ significantly reducing tumor volumes, the invasive margins of tumors were not affected (Fig. 5). However, combinatorial treatment with PF-562271 resulted in a significant reduction in invasive margins. Additionally, a reduction in tumor volumes and the Ki67 proliferation index, an increase in apoptosis, and an increase in animal survival rates make TMZ and PF-562271 combinatorial treatment a potential strategy for GBM. The efficacy of Pyk2/FAK inhibitors has been demonstrated in various preclinical studies and nonbrain tumors [15, 40, 41]. However, human clinical trials identified only modest progression-free survival in patients receiving FAK/Pyk2 inhibitors individually [42, 43]. Nevertheless, the results obtained from this study suggest that combinatorial treatment can be a future medical approach for GBM and is a subject for human clinical trials.

## Conclusion

TMZ increases Pyk2 and FAK phosphorylation and Bcl2 expression in GBM cells. These effects are reversed by PF-562271. PF-562271, given in combination with TMZ, reduced cell viability, cell cycle progression, invasion and invadopodia activity compared with TMZ alone. In vivo studies demonstrated that combinatorial treatment led to a prominent reduction in tumor size and invasive margins, a reduction in the Ki67 proliferation index, and an increase in apoptosis and animal survival rates compared with TMZ monotherapy.

## Supplementary Information

Below is the link to the electronic supplementary material.

Supplementary file1 (PDF 1444 kb)

## Data Availability

The data generated in this study are available within the article.
