# Supplementary material for: The PYK2 inhibitor PF-562271 enhances the effect of temozolomide on tumor growth in a C57Bl/6-Gl261 mouse glioma model
Source: J Neurooncol. 2023 Feb 15;161(3):593–604. doi: 10.1007/s11060-023-04260-3 (PMC9992029; doi:10.1007/s11060-023-04260-3)
Supplement: Supplementary file 1 — Supplementary file1 (PDF 1444 kb) [file 11060_2023_4260_MOESM1_ESM.pdf]

The PYK2 inhibitor PF-562271 enhances the effect of temozolomide on tumor growth in a C57Bl/6-Gl261 mouse glioma model

Journal of Neuro-Oncology

Jescelica Ortiz-Rivera[1], Rebeca Nunez[1], Yuriy Kucheryavykh[1], Lilia Kucheryavykh[1]*

1 Department of Biochemistry, Universidad Central de Caribe, School of Medicine, Bayamon, PR, 00956; 416jortiz@uccaribe.edu, rebeca.nunez@upr.edu, yuriy.kucheryavykh@uccaribe.edu, lilia.kucheryavykh@uccaribe.edu

* Correspondence: 416jortiz@uccaribe.edu

Cells and Antibodies	RRID
GL261	(NCI-DTP Cat# Glioma 261, RRID:CVCL_Y003)
HMC3	(ATCC Cat# CRL-3304, RRID:CVCL_II76)
SIM-A9	(ATCC Cat# CRL-3265, RRID:CVCL_5I31)
Pyk2	(Cell Signaling Technology Cat# 3480, RRID:AB_2174093)
phospho-Pyk2 (Tyr 579/580)	(Thermo Fisher Scientific Cat# 44-636G, RRID:AB_2533706)
FAK	(Cell Signaling Technology Cat# 3285, RRID:AB_2269034)
phospho-FAK (Tyr 925)	(Cell Signaling Technology Cat# 3284, RRID:AB_10831810)
KI67	(Cell Signaling Technology Cat# 12075, RRID:AB_2728830)
BCL2	(Cell Signaling Technology Cat# 3498, RRID:AB_1903907)
Cyclin D1	(Cell Signaling Technology Cat# 55506, RRID:AB_2827374)
MGMT	(Cell Signaling Technology Cat# 2739, RRID:AB_2297658)

Online Recourse 1: Research Resource Identifiers for cells and antibodies.

The PYK2 inhibitor PF-562271 enhances the effect of temozolomide on tumor growth in a C57Bl/6-Gl261 mouse glioma model

Journal of Neuro-Oncology

Jescelica Ortiz-Rivera[1], Rebeca Nunez[1], Yuriy Kucheryavykh[1], Lilia Kucheryavykh[1]*

2 Department of Biochemistry, Universidad Central de Caribe, School of Medicine, Bayamon, PR, 00956; 416jortiz@uccaribe.edu, rebeca.nunez@upr.edu, yuriy.kucheryavykh@uccaribe.edu, lilia.kucheryavykh@uccaribe.edu

* Correspondence: 416jortiz@uccaribe.edu

a

Cell lines	Adherence	sex	NF1	MGMT	IDH
CL-2	adherent	M	+	+	WT
CL-3	adherent	F	-	+	WT

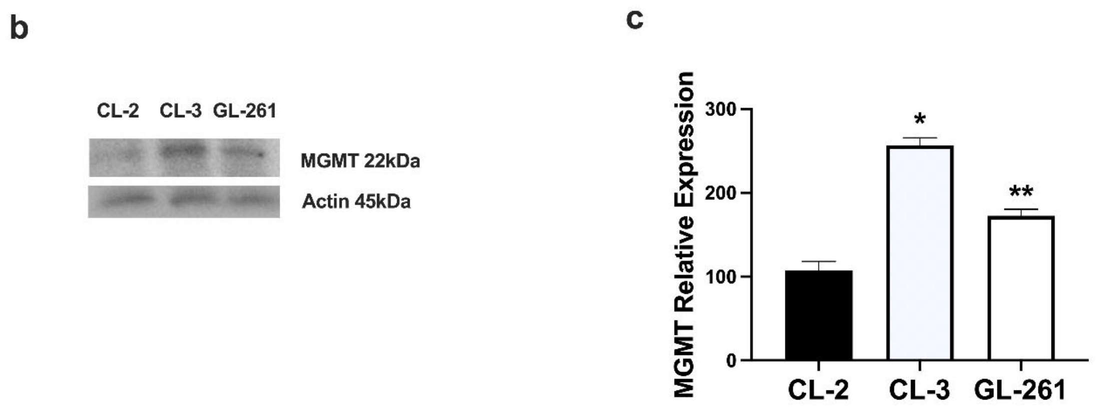

Online Recourse 2: Characterization of human GBM derived primary cells (CL2 and CL3). Patient data and key signaling proteins expression data, detected by RT-PCR and western blot (a). Representative western blots and quantitative results for O[6]-methylguanine-DNA methyltransferase (MGMT) are presented for CL-2, CL-3 and GL261 cells (b). The values are shown as means ± SD of three repetitions per cell line.

The PYK2 inhibitor PF-562271 enhances the effect of temozolomide on tumor growth in a C57Bl/6-Gl261 mouse glioma model

Journal of Neuro-Oncology

Jescelica Ortiz-Rivera[1], Rebeca Nunez[1], Yuriy Kucheryavykh[1], Lilia Kucheryavykh[1]*

3 Department of Biochemistry, Universidad Central de Caribe, School of Medicine, Bayamon, PR, 00956; 416jortiz@uccaribe.edu, rebeca.nunez@upr.edu, yuriy.kucheryavykh@uccaribe.edu, lilia.kucheryavykh@uccaribe.edu

* Correspondence: 416jortiz@uccaribe.edu

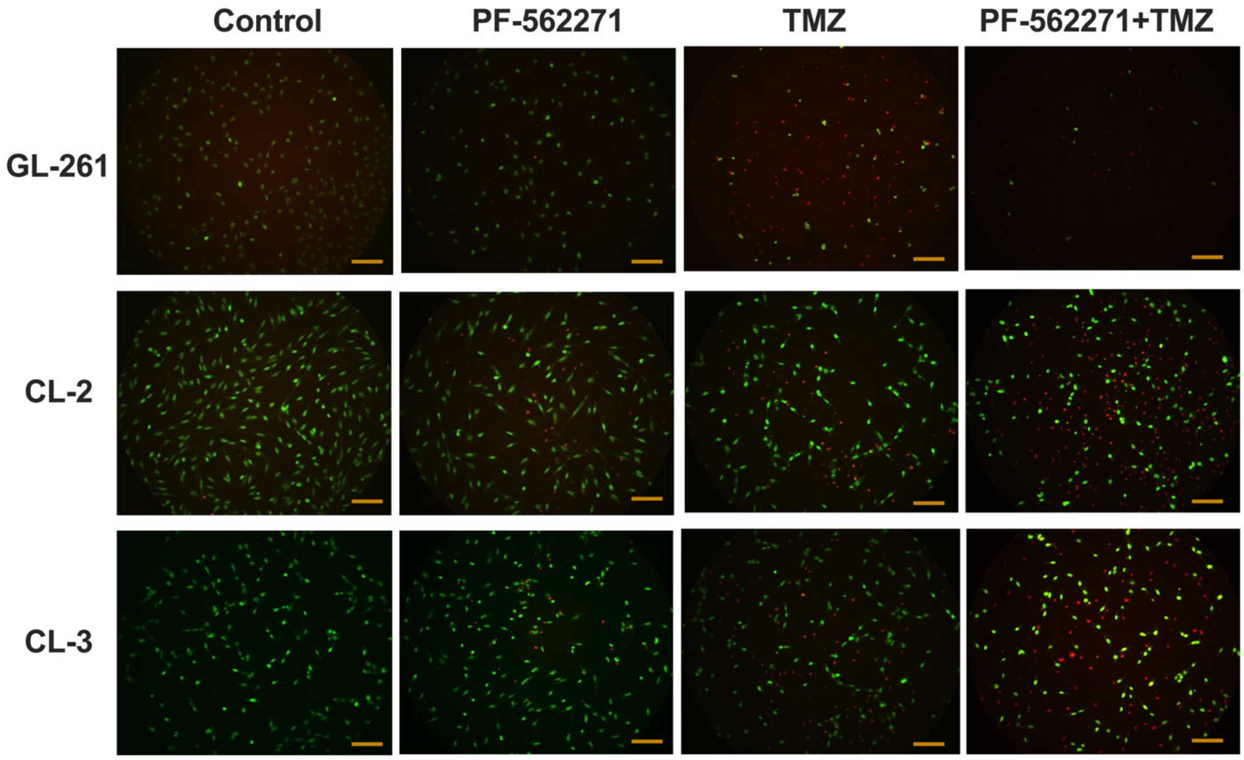

Online Recourse 3: Representative fluorescence images for live-dead viability assays are presented in support of Figure 2 a-c. Calcein (green, live cells) and ethidium homodimer-1 (red, dead cells) staining after 72 hours of treatment with vehicle (control), PF-562271, TMZ, and PF-562271+TMZ in GL261, CL-2, and CL-3 cell lines is shown. Scale bar: 80 µm.

The PYK2 inhibitor PF-562271 enhances the effect of temozolomide on tumor growth in a C57Bl/6-Gl261 mouse glioma model

Journal of Neuro-Oncology

Jescelica Ortiz-Rivera[1], Rebeca Nunez[1], Yuriy Kucheryavykh[1], Lilia Kucheryavykh[1]*

4 Department of Biochemistry, Universidad Central de Caribe, School of Medicine, Bayamon, PR, 00956; 416jortiz@uccaribe.edu, rebeca.nunez@upr.edu, yuriy.kucheryavykh@uccaribe.edu, lilia.kucheryavykh@uccaribe.edu

* Correspondence: 416jortiz@uccaribe.edu

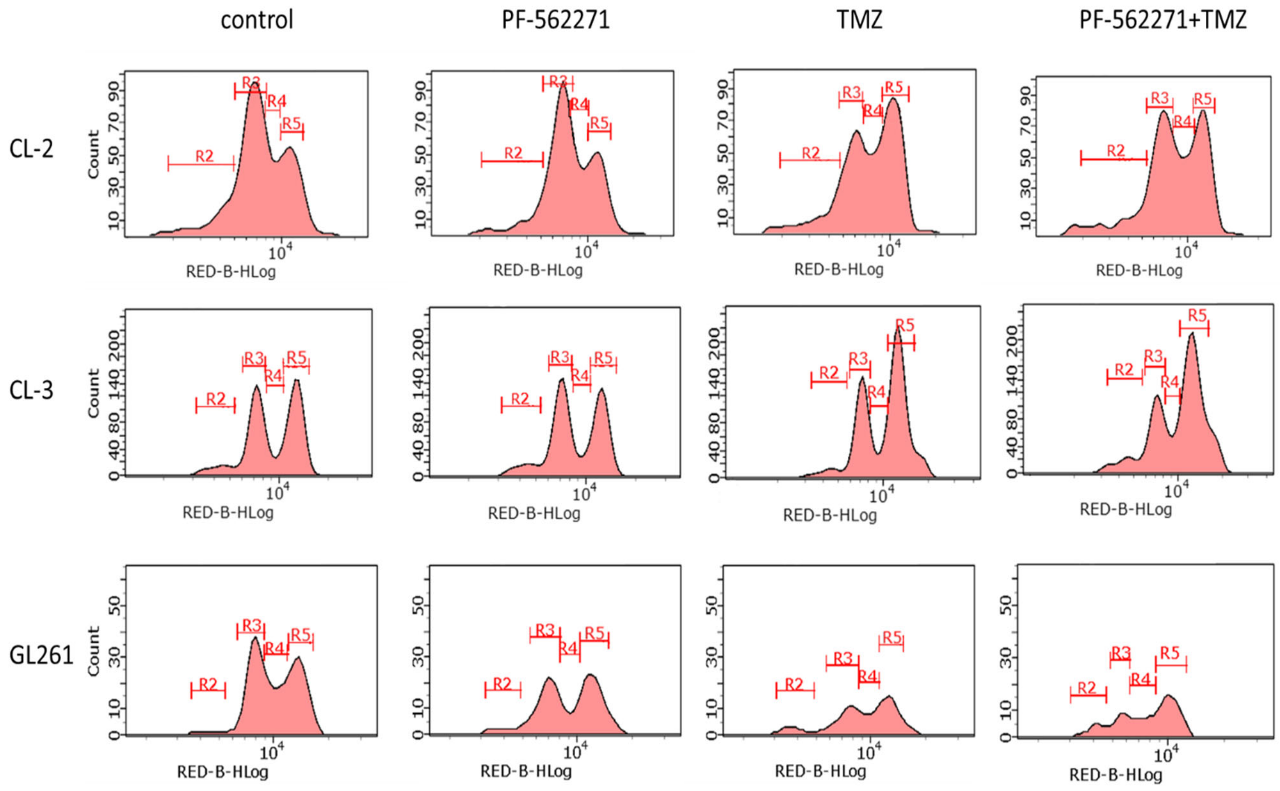

Online resource 4: Representative histograms for cell cycle analysis, using flow cytometric evaluation of propidium iodide as a nuclear marker in support of Fig 2 d-f. Study performed for CL-2, CL-3 and GL261 cells, treated for 72 hours with vehicle, TMZ, PF-562271, and TMZ and PF-562271 in combination. Fractions of cells in the sub-G1 (R2), G0/G1 (R3), S (R4) and G2/M (R5) phases was determined based on DNA content.

The PYK2 inhibitor PF-562271 enhances the effect of temozolomide on tumor growth in a C57Bl/6-Gl261 mouse glioma model

Journal of Neuro-Oncology

Jescelica Ortiz-Rivera[1], Rebeca Nunez[1], Yuriy Kucheryavykh[1], Lilia Kucheryavykh[1]*

5 Department of Biochemistry, Universidad Central de Caribe, School of Medicine, Bayamon, PR, 00956; 416jortiz@uccaribe.edu, rebeca.nunez@upr.edu, yuriy.kucheryavykh@uccaribe.edu, lilia.kucheryavykh@uccaribe.edu

* Correspondence: 416jortiz@uccaribe.edu

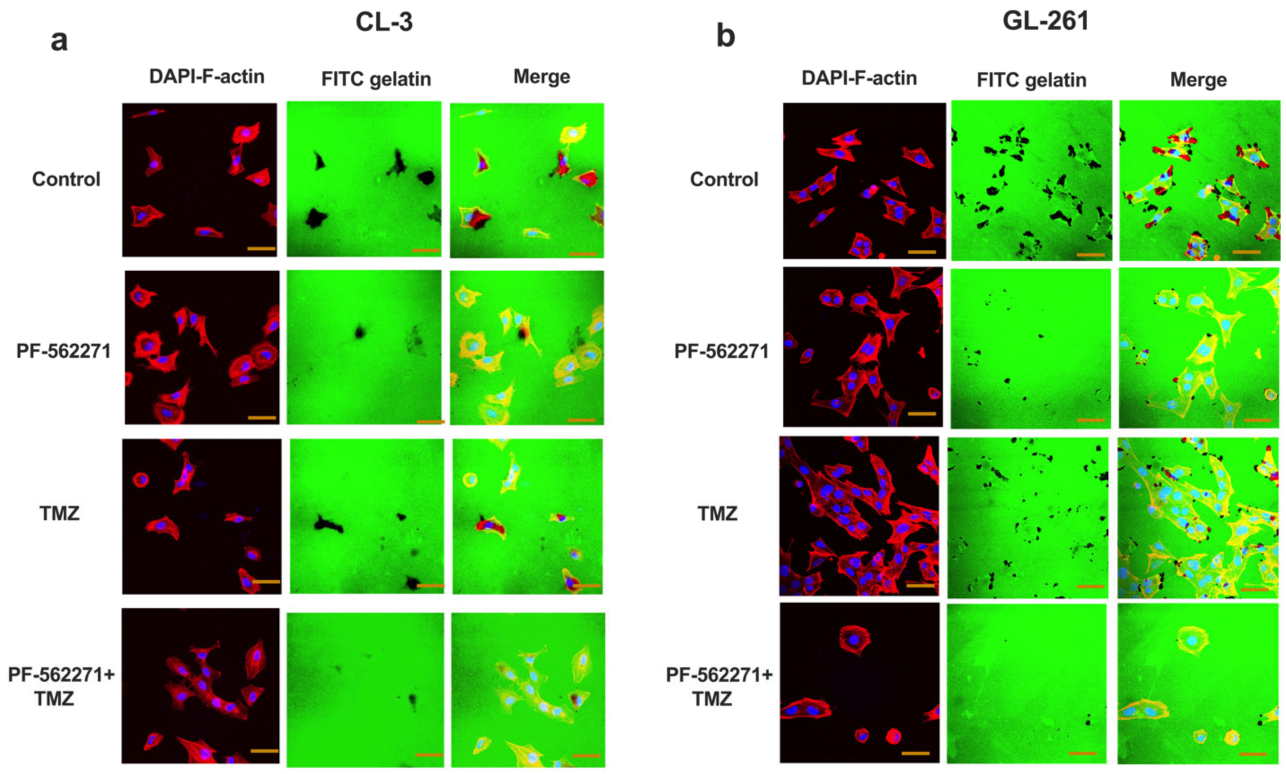

Online Recourse 5: Representative confocal images for invadopodia formation assays for CL-3 (a) and GL261(b) cells, treated with vehicle, TMZ, PF-562271, and TMZ and PF-562271 in combination, are presented in support of Figure 4 c, d, f, g. F-actin, stained with rhodamine–phalloidin (red), FITC-conjugated gelatin (green), and DAPI, used for nuclei staining (blue), are shown. Degraded areas of FITC-labeled gelatin are shown as black patches. Study duration was 16 hours. Scale bar: 60 µm.
